# Attenuated inflammatory profile following single and repeated handgrip exercise and remote ischemic preconditioning in patients with cerebral small vessel disease

**DOI:** 10.3389/fphys.2022.1026711

**Published:** 2022-11-21

**Authors:** Thijs R. J. Landman, Laween Uthman, Inge A. H. Hofmans, Yvonne Schoon, Frank-Erik de Leeuw, Dick H. J. Thijssen

**Affiliations:** ^1^ Departmenet of Physiology, Radboud Institute for Health Sciences, Radboud University Medical Centre, Gelderland, Netherlands; ^2^ Departmenet of Geriatric Medicine, Radboud Institute for Health Sciences, Radboud University Medical Centre, Gelderland, Netherlands; ^3^ Center for Cognitive Neuroscience, Department of Neurology, Donders Institute for Brain, Cognition and Behaviour, Radboud University Medical Centre, Gelderland, Netherlands

**Keywords:** exercise, remote ischemic preconditioning, cerebral small vessel disease, inflammation, proteomics

## Abstract

**Background:** Similar to remote ischemic preconditioning bouts of exercise may possess immediate protective effects against ischemia-reperfusion injury. However, underlying mechanisms are largely unknown. This study compared the impact of single and repeated handgrip exercise versus remote ischemic preconditioning on inflammatory biomarkers in patients with cerebral small vessel disease (cSVD).

**Methods:** In this crossover study, 14 patients with cSVD were included. All participants performed 4-day of handgrip exercise (4x5-minutes at 30% of maximal handgrip strength) and remote ischemic preconditioning (rIPC; 4x5-minutes cuff occlusion around the upper arm) twice daily. Patients were randomized to start with either handgrip exercise or rIPC and the two interventions were separated by > 9 days. Venous blood was drawn before and after one intervention, and after 4-day of repeated exposure. We performed a targeted proteomics on inflammation markers in all blood samples.

**Results:** Targeted proteomics revealed significant changes in 9 out of 92 inflammatory proteins, with four proteins demonstrating comparable time-dependent effects between handgrip and rIPC. After adjustment for multiple testing we found significant decreases in FMS-related tyrosine kinase-3 ligand (Flt3L; 16.2% reduction; adjusted *p*-value: 0.029) and fibroblast growth factor-21 (FGF-21; 32.8% reduction adjusted *p*-value: 0.029) after single exposure. This effect did not differ between handgrip and rIPC. The decline in Flt3L after repeated handgrip and rIPC remained significant (adjusted *p*-value = 0.029), with no difference between rIPC and handgrip (adjusted *p*-value = 0.98).

**Conclusion:** Single handgrip exercise and rIPC immediately attenuated plasma Flt3L and FGF-21, with the reduction of Flt3L remaining present after 4-day of repeated intervention, in people with cSVD. This suggests that single and repeated handgrip exercise and rIPC decrease comparable inflammatory biomarkers, which suggests activation of shared (anti-)inflammatory pathways following both stimuli. Additional studies will be needed to exclude the possibility that this activation is merely a time effect.

## Introduction

Ischemia-reperfusion (I/R)-injury represents a contributor to cerebral- and cardiovascular-related morbidity and mortality (Hausenloy et al., 2017; Heusch, 2020). While reperfusion is essential for restoring blood flow to tissue and preventing tissue necrosis from ischemia (e.g., during an infarction or surgery), the rapid reintroduction of blood flow paradoxically causes additional injury. In addition to timely reperfusion previous studies revealed that remote ischemic preconditioning (rIPC) may reduce this I/R-injury. rIPC refers to the exposure of remote tissue (e.g., a limb) to repeated periods of short-lasting ischemia (e.g., 4 times 5-min) (Hess et al., 2015) which improves resilience to a period of prolonged ischemia in a target organ (e.g., the brain and heart) (Przyklenk et al., 1993; Benstoem et al., 2017). Despite the success of pre-clinical studies, a series of clinical trials evaluating the efficacy of rIPC (Hausenloy et al., 2015; Meybohm et al., 2018; Hausenloy et al., 2019) failed to translate this into improved clinical outcome. It is not exactly known by which mechanisms preconditioning might work. Previous studies suggest that rIPC causes the release of humoral factors that can affect the target organ (e.g., heart or brain) *via* the circulation (Iadecola and Anrather, 2011). These humoral factors possibly relate to inflammatory biomarkers.

Interestingly, within the past few years, a series of studies in animals and humans have suggested that exercise also induces immediate preconditioning effects (Seeger et al., 2015; Thijssen et al., 2018). Whether exercise and rIPC share underlying pathways related to the possibility to protect cerebral and cardiac tissue against I/R-injury is largely unknown (Thijssen et al., 2018). Exercise has been shown to also influence inflammatory pathways, but the molecular effects (e.g., inflammatory, metabolic, cardiovascular and immune pathways) are very complex (Contrepois et al., 2020). This makes it likely that exercise also activates (inflammatory) pathways that are not influenced by rIPC. Insight into this hypothesis will help to understand the mechanisms underlying the effects of exercise, but also to understand the overlap, or lack thereof, between exercise and rIPC in activation of inflammatory pathways. Understanding these mechanisms could help guide the translation of remote ischemic preconditioning to clinical application.

Whilst the majority of studies have focused on the impact of single exposure to rIPC, little work explored whether repeated bouts of preconditioning may be more effective than single application. Repeated exposure to preconditioning may enhance the efficacy of the preconditioning stimulus (Thijssen et al., 2016), possibly through combining the effects of the early-phase (1–2 h post-preconditioning) and late-phase protection (starting 12–72 h after the stimulus and persists for days to weeks) (Dirnagl et al., 2009). Therefore, the purpose of this study was to compare the impact of single and repeated handgrip exercise versus remote ischemic preconditioning on inflammatory biomarkers (using proteomics analysis) in patients with cerebral small vessel disease (cSVD). For this purpose, we selected cSVD since this patient group has an increased risk for cerebro-cardiovascular events (Rost and Etherton, 2020; Suzuyama et al., 2020) and shows increased susceptibility to injury following cerebral I/R (Leech et al., 2019). We performed proteomics analysis to compare the release of blood-borne markers of inflammation between exercise and rIPC. We hypothesised that both exercise and rIPC alter inflammatory biomarkers, with strong overlap between both stimuli, which would highlight the potential of exercise as a preconditioning stimulus.

## Materials and methods

### Study population and design

In this cross-over intervention study, we included 14 patients with clinical symptoms of cSVD (i.e., lacunar stroke or transient ischemic attack, cognitive disturbances, motor disturbances and/or depressive symptoms) with accompanying MRI markers of cSVD from the Radboud University Nijmegen Diffusion tensor and Magnetic resonance imaging Cohort (RUNDMC) study, which is a prospective study that investigated risk factors and clinical consequences of cSVD. The full protocol of the RUNDMC study has been described previously (van Norden et al., 2011). Patients were selected from the participants that completed the most recent follow-up measurements of the RUNDMC study in 2021. Inclusion criteria were: 1) age above 60 years, 2) mentally able to give informed consent. Exclusion criteria were: 1) upper extremity injury or edema contra-indicating handgrip exercise or rIPC, 2) mastectomy on both sides. All patients signed informed consent before participating. The study was approved by the ethical committee (CMO Arnhem-Nijmegen, registration number 2020-6781).

### Intervention

All participants underwent the handgrip exercise and rIPC interventions with at least 9-day wash-out period in between and were randomly assigned to start with either handgrip or rIPC. Handgrip involved a protocol of 4 cycles of 5 min of handgrip exercise, divided by 5 min of rest. To determine the setting for the dynamometer maximal handgrip strength was assessed during screening. The dynamometer was thereafter set at 30% of maximal handgrip strength and grip and relaxation were alternated every second. rIPC was performed by inflating a blood pressure cuff around the upper arm at 200 mmHg (after making sure that no patient had a blood pressure that exceeded 180 mmHg). The rIPC intervention was performed in 4 cycles of 5 min with 5 min of reperfusion in between.

The first intervention cycle of both the handgrip and rIPC took place at the department of Physiology, Radboudumc, under supervision of a trained researcher (a research physician). This was always performed on Monday morning and timing was consistent for both interventions within each individual participant. Thereafter, participants independently continued the intervention at home twice daily for 4 days, with at least 6 hours of rest between the two interventions each day. On the morning of the fifth day (Friday), the participants again visited the Radboudumc to perform the last intervention, resulting in 9 cycles per participant over the course of 5 days.

### Blood sampling

**FIGURE 1:**
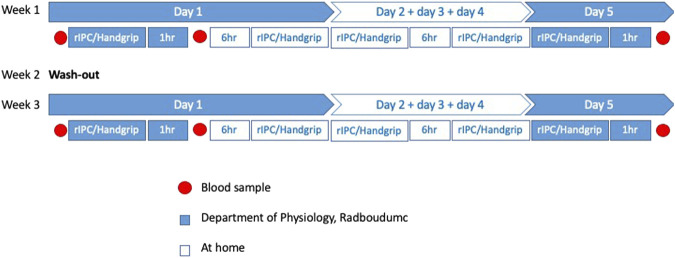
Overview study design. An overview of all the measurements in this cross-over study. rIPC = Remote ischemic preconditioning.

Venous blood was drawn at three time points in both intervention weeks: 1) baseline (directly before the start of the first intervention), 2) 1 hour after completion of the first intervention, and 3) 1 hour after the last intervention on day 5, resulting in 6 samples per patient: rIPC baseline, rIPC single, rIPC repeated, Handgrip baseline, Handgrip single and Handgrip repeated. Timing of blood sampling was consistent for both intervention weeks within each individual patient. Figure 1 represents an overview of the study design. At each time point 40 ml of venous blood was drawn and was collected in lithium-heparin tubes. The samples were centrifuged at 1.400 RCF for 10 min at 4°C. The plasma (−20 ml per sample) was collected and stored at −80°C.

### Proteomics analysis

As a primary outcome the effect of handgrip and rIPC on inflammatory biomarkers was explored. For the proteomics analysis all 84 samples were analyzed simultaneously for 92 unique protein biomarkers using a pre-designed Proseek Multiplex® immunoassay panel; Inflammation (OLINK Proteomics, Uppsala, Sweden). The entire list of proteins can be found in Supplementary Table S1. Plasma was analyzed using Proximity Extension Assay (PEA), which has a high specificity and sensitivity (Assarsson et al., 2014). Validation data and limits of detection (LOD) are available at the manufacturer’s webpage (http://www.olink.com). The proteomics data have been deposited to the ProteomeXchange Consortium *via* the PRIDE (Perez-Riverol et al., 2022) partner repository with the dataset identifier PXD037267.

The Olink assay was performed with a fixed proportion of the plate designated for control samples. Samples were randomly allocated to wells. The outcome data was normalized using standard Olink workflows to produce relative protein abundance on a log2 scale (‘NPX’). Quality assessment was performed by examination of Olink internal controls. Following this step, one sample with poor-quality was removed. 37 proteins had more than 75% of the samples below the limit of detection and were excluded from the final analysis. For the remaining 55 proteins, the change in NPX for each protein between baseline and after single application was determined and compared between rIPC and handgrip. Additionally, the difference in NPX levels for each inflammatory biomarker after repeated preconditioning was analyzed for both rIPC and handgrip.

### Statistical analysis

Analyses were performed using RStudio (TEAM, 2019) (Team, 2019). All outcomes were checked for normality. The differences in NPX for each protein were investigated with two linear mixed models (baseline *versus* single and baseline *versus* repeated). Linear mixed models were performed using the lmer function in the lme4 package (BATES et al., 2015) with a random intercept. The fixed variables were time (baseline, single and repeated) and treatment (rIPC and handgrip), including an interaction term for time*treatment. We used the Benjamini–Hochberg method to correct for multiple testing of the 55 inflammatory biomarkers. Adjusted *p*-values <0.05 were considered statistically significant, but because of the exploratory nature of this study, we also reported all proteins that had unadjusted *p*-values <0.05.

## Results

Fourteen cSVD patients participated. Their mean age was 70.4 ± 3.6 years and 57% was male. All baseline characteristics are presented in Table 1.

**TABLE 1:** Baseline characteristic.

Table 1. Baseline characteristics	Total **(N = 14)**
**Age (years)**	70.4 (±3.6)
**Sex, male n (%)**	8 (57.1%)
**BMI (m/kg** ^**2**^ **)**	24.7 (±3.9)
**Blood pressure**	
**Systolic (mmHg)**	142 (±17)
**Diastolic (mmHg)**	81 (±9)
**Medical history, n (%)**	
**Atrial fibrillation**	1 (7.1)
**Hypertension**	8 (57.1)
**TIA**	2 (14.3)
**Stroke**	2 (14.3)
**Diabetes Mellitus**	1 (7.1)
**Alcohol use (yes), n (%)**	11 (78.6)
**Smoking status, n (%)**	
**Never**	2 (14.3)
**Former**	11 (78.6)
**Active**	1 (7.1)
**Max handgrip strength (kg)**	32.4 (±11.5)
**MRI markers of cSVD**	
**White matter hyperintensity volume (ml)**	5.1 [1.2, 43.5]
**Lacunes (n)**	0 [0,2]
**Microbleeds (n)**	0 [0,1]

Values are mean (± standard deviation), number (percentage) or median [min, max]. Abbreviations: TIA, Transient ischemic attack; BMI, body mass index.

**FIGURE 2:**
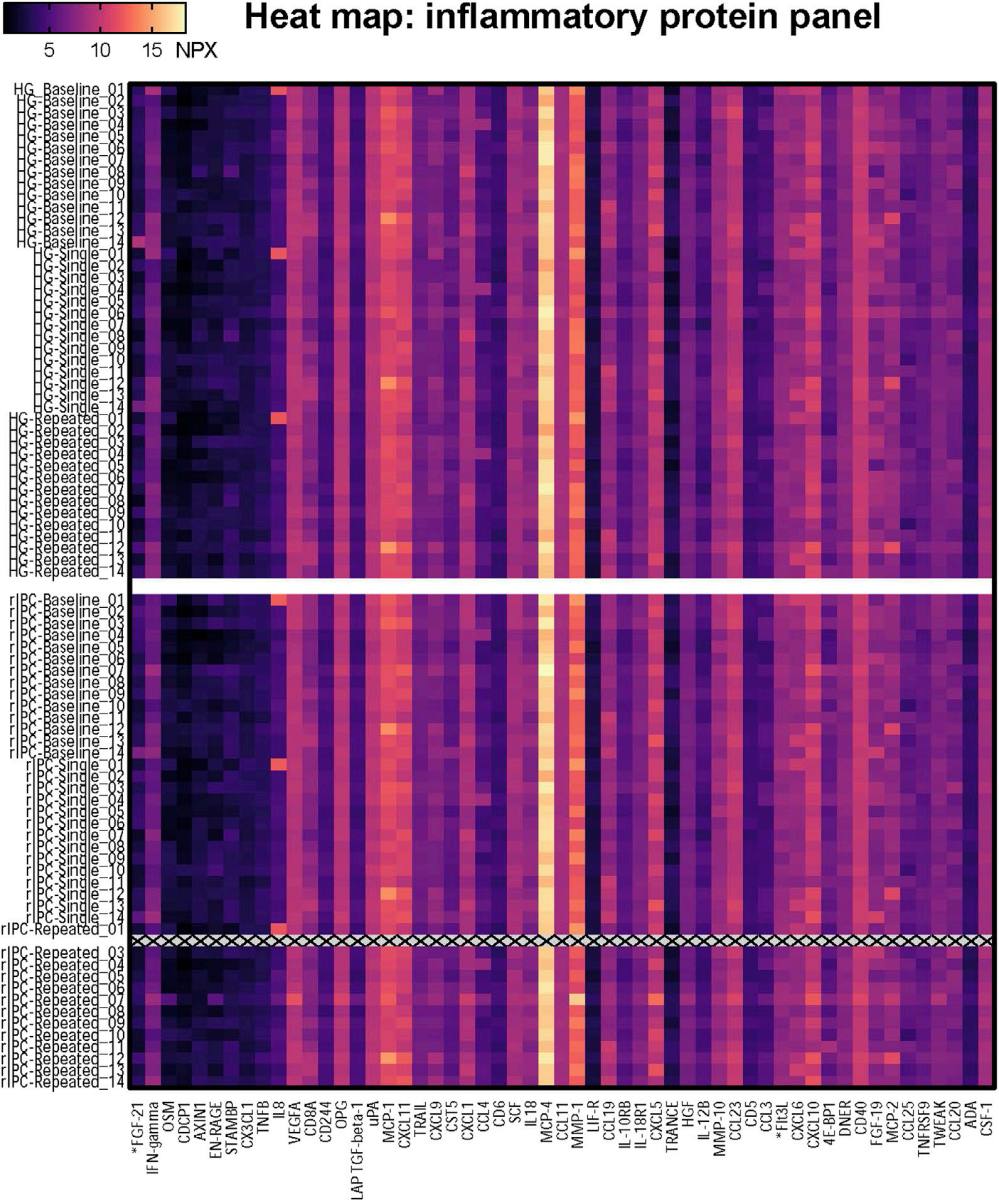
Heatmap of the proteomics analysis. The heatmap shows the NPX value for the 55 proteins in each individual sample; 14 participants who underwent two conditionings (Handgrip and rIPC) on three timepoints (baseline, single, repeated). HG = Handgrip; rIPC = Remote ischemic preconditioning.

**FIGURE 3:**
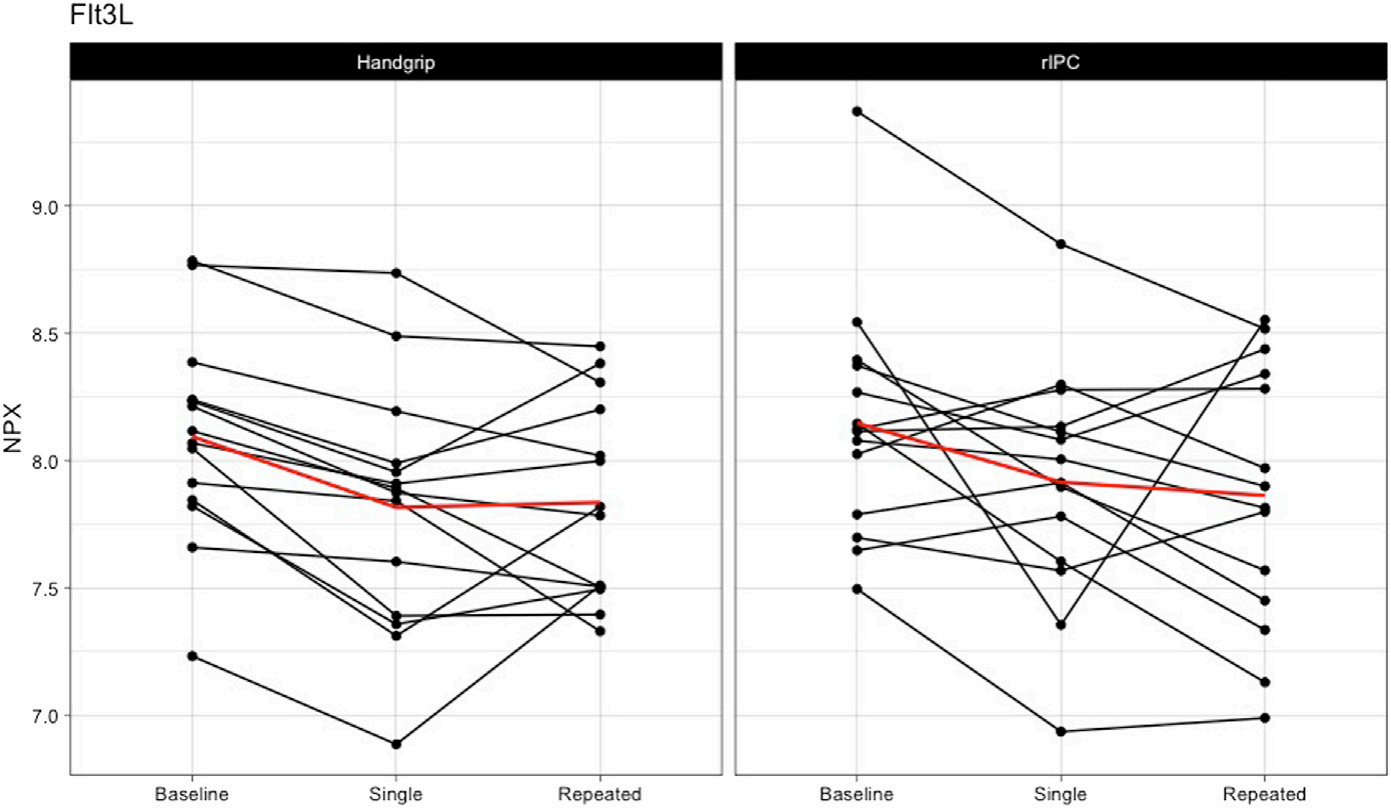
Changes in Flt3L after single and repeated preconditioning. The changes in the NPX of Flt3L (on the y-axis) between baseline and after single and repeated intervention (on the x-axis) for both the handgrip intervention (on the left) and the remote ischemic preconditioning (on the right). Each black line represents one participant and the red line is the mean for all the participants together. rIPC = Remote ischemic preconditioning. NPX = Normalised protein expression; Flt3L = FMS-related tyrosine kinase 3 ligand.

**FIGURE 4:**
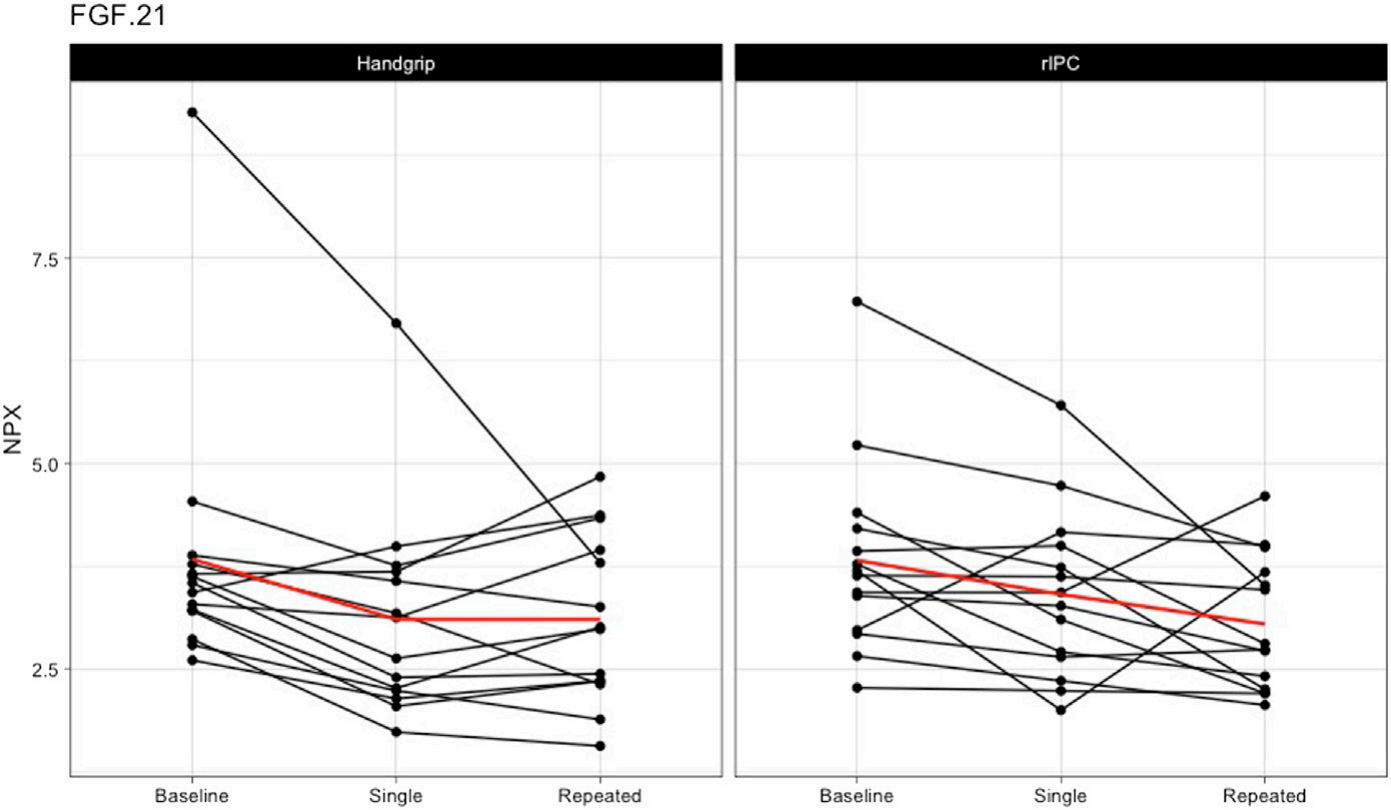
Changes in FGF-21 after single and repeated preconditioning. The changes in the NPX of FGF-21 (on the y-axis) between baseline and after single and repeated intervention (on the x-axis) for both the handgrip intervention (on the left) and the remote ischemic preconditioning (on the right). Each black line represents one participant and the red line is the mean for all the participants together. rIPC = Remote ischemic preconditioning. NPX = Normalised protein expression; FGF-21 = fibroblast growth factor 21.

*Single exercise and rIPC.* The mixed model analysis showed a significant main effect in 9 (out of 55) proteins (Table 2; Figure 2) after single intervention. We found five proteins with a significant reduction over time (before versus after single exposure: Flt3L, FGF-21, TRANCE, MMP-1, and MCP-1) and five proteins with a significant difference between exercise and rIPC (TRANCE, CCL19, CCL11, CXCL5, and CD6). There were no significant interaction effects. After adjustment for multiple testing, we found significant decreases in FMS-related tyrosine kinase 3 ligand (Flt3L) and fibroblast growth factor 21 (FGF-21). The NPX for Flt3L reduced from 8.12 to 7.87 (16.2% reduction on a linear scale; adjusted *p*-value: 0.029; Figure 3) and FGF-21 from 3.83 to 3.25 (32.8% reduction on a linear scale; adjusted *p*-value: 0.029; Figure 4). We found no difference in changes in Flt3L and FGF-21 between handgrip and rIPC (adjusted *p*-value: 0.86 and 0.91, respectively).

**TABLE 2:** Levels of inflammatory biomarkers before and after single preconditioning.

	Handgrip		rIPC		*p*-values (unadjusted)			*p*-values (adjusted)		
	Baseline	Single	Baseline	Single	Treatment	Time	Time*Treatment	Treatment	Time	Time*Treatment
Flt3L	8.09 ± 0.41	7.82 ± 0.49	8.15 ± 0.46	7.91 ± 0.46	0.24	**<0.001**	0.72	0.86	**0.029**	0.79
FGF-21	3.83 ± 1.64	3.10 ± 1.27	3.82 ± 1.18	3.41 ± 1.03	0.33	**<0.001**	0.29	0.91	**0.029**	0.86
TRANCE	2.95 ± 0.66	2.71 ± 0.65	3.15 ± 0.64	2.92 ± 0.55	**0.004**	**0.001**	0.98	0.18	0.06	0.99
CCL19	8.18 ± 0.97	8.16 ± 1.05	8.34 ± 0.87	8.37 ± 0.99	**0.02**	0.94	0.76	0.57	0.97	0.97
MMP-1	12.51 ± 0.75	12.41 ± 0.85	12.70 ± 0.89	12.42 ± 0.96	0.24	**0.03**	0.30	0.86	0.71	0.87
MCP-1	11.52 ± 0.82	11.28 ± 0.92	11.55 ± 0.89	11.43 ± 0.89	0.26	**0.03**	0.51	0.86	0.73	0.96
CCL11	8.63 ± 0.14	8.60 ± 0.16	8.66 ± 0.18	8.67 ± 0.11	**0.044**	0.74	0.44	0.73	0.97	0.96
CXCL5	9.88 ± 0.80	9.85 ± 0.69	10.19 ± 0.74	10.01 ± 0.84	**0.047**	0.38	0.50	0.73	0.94	0.96
CD6	4.14 ± 0.32	4.08 ± 0.47	4.23 ± 0.30	4.24 ± 0.32	**0.049**	0.70	0.57	0.73	0.97	0.96

The bold values represent statistically significant results.

Values are presented as mean NPX (Normalised protein expression; relative protein abundance on a log2 scale) ± standard deviation for each protein on baseline and after single handgrip/rIPC., Linear mixed model factors: Treatment, Time and Time*Treatment (unadjusted and adjusted).

*Repeated exercise and rIPC.* Analysis comparing baseline with repeated handgrip exercise or repeated rIPC largely supported the findings of the initial analysis, in that we found a significant change in 7 out of the 55 proteins (table 3). After adjustment for multiple testing, the decline in Flt3L after repeated handgrip and rIPC remained significant (NPX for Flt3L reduced from 8.12 to 7.84 (17.2% reduction on a linear scale; adjusted *p*-value: 0.029; Figure 3), with no difference between rIPC and handgrip (adjusted *p*-value: 0.98). We found no difference between the effect size of single versus repeated handgrip exercise or rIPC on Flt3L (adjusted *p*-value: 0.95) or on any other inflammatory biomarker.

**TABLE 3:** Levels of inflammatory biomarkers before and after repeated preconditioning.

	Handgrip		rIPC		*p*-values (unadjusted)			*p*-values (adjusted)		
	Baseline	Repeated	Baseline	Repeated	Treatment	Time	Time*Treatment	Treatment	Time	Time*Treatment
Flt3L	8.09 ± 0.41	7.84 ± 0.39	8.15 ± 0.46	7.86 ± 0.52	0.54	**<0.001**	0.85	0.98	**0.029**	0.99
FGF-21	3.83 ± 1.64	3.10 ± 1.01	3.82 ± 1.18	3.04 ± 0.82	0.87	**0.002**	0.93	0.99	0.13	0.99
CCL20	6.96 ± 0.70	6.66 ± 0.64	6.66 ± 0.63	6.99 ± 0.74	0.92	0.90	**0.01**	0.99	0.99	0.63
MCP-1	11.52 ± 0.82	11.31 ± 0.99	11.55 ± 0.89	11.40 ± 0.99	0.44	**0.04**	0.74	0.98	0.98	0.99
CXCL5	9.88 ± 0.80	9.80 ± 0.51	10.19 ± 0.74	10.10 ± 1.22	**0.046**	0.57	0.96	0.98	0.98	0.99
IFN-gamma	6.75 ± 1.03	6.76 ± 0.69	6.53 ± 0.89	6.39 ± 1.07	**0.047**	0.66	0.63	0.98	0.98	0.98
TRANCE	2.95 ± 0.66	2.85 ± 0.62	3.15 ± 0.64	2.96 ± 0.64	**0.047**	0.06	0.54	0.98	0.98	0.98

The bold values represent statistically significant results.

Values are presented as mean NPX (Normalised protein expression; relative protein abundance on a log2 scale) ± standard deviation for each protein on baseline and after repeated handgrip/rIPC., Linear mixed model factors: Treatment, Time and Time*Treatment (unadjusted and adjusted).

## Discussion

The purpose of our study was to examine the impact of single and repeated (handgrip) exercise and rIPC on inflammatory biomarkers through targeted proteomics analysis. Targeted proteomics revealed a significant reduction in various inflammatory proteins upon exposure to single handgrip or rIPC, with no further changes thereafter. Changes in inflammatory proteins following handgrip exercise and rIPC were in part overlapping, with most prominent reductions in the pro-inflammatory biomarkers Flt3L and FGF-21 after both interventions. Despite this overlap, some proteins demonstrated a distinct pattern between handgrip and rIPC. This suggests that, at least partly, there is overlap in inflammatory biomarkers in response to (repeated) bouts of exercise and ischemia as preconditioning stimuli in patients with cSVD.

In line with our hypothesis, we found that a single bout of handgrip exercise has immediate effects on inflammatory biomarkers. This effect of a single bout of handgrip exercise was in line with rIPC, which supports the concept that exercise, similar to rIPC, has preconditioning effects (Thijssen et al., 2018). Interestingly, previous studies reported reduced efficacy of rIPC in older patients with endothelial dysfunction (Seeger et al., 2016; van den Munckhof et al., 2013). As patients with cSVD chronically suffer from damage to the smaller vessels of the brain due to endothelial dysfunction (Quick et al., 2021), we expected an attenuated or even absent efficacy of rIPC compared to exercise. Although we have not compared the effects in patients with cSVD with healthy controls and are therefore unable to provide insight into a potentially attenuated efficacy of a single bout of preconditioning, our results suggest that exercise and rIPC both have immediate effects on inflammatory protein expression in this population of older patients with cSVD. One possible reason for this observation is selection bias, consequently resulting in the inclusion of a relatively healthy, physically active, population of cSVD patients. In a small proof-of-concept study, physical activity indeed restored the efficacy of ischemic preconditioning in senescent rat hearts (Abete et al., 2000). Similarly, in previous work we have demonstrated that regular physical activity has beneficial effects, and may restore preconditioning effects in older individuals or patients with heart failure (Seeger et al., 2016; Seeger et al., 2015; van den Munckhof et al., 2013). At least, our data indicate the ability of handgrip exercise and rIPC to influence inflammatory protein expression in a population of cSVD patients. Similarly, the inclusion of a relatively healthy group may also explain why repeated exposure does not further increase the magnitude of this response.

Findings from our explorative proteomics analysis identified reduction of Flt3L after handgrip exercise and rIPC. Flt3L may play a role in development of cardiovascular disease as upregulation of Flt3L leads to dendritic cell development, subsequently mediating blood pressure elevation and development of atherosclerosis (Zernecke, 2015; Lu et al., 2020). Similarly, a previous study found increased Flt3 expression following I/R-injury in a human neuroblastoma cell line (Dong et al., 2019), whilst silencing of Flt3 prevented I/R-injury in these cells, suggesting a mediating role for Flt3 during I/R-injury (Dong et al., 2019). Our study is the first to show a decrease in Flt3L after exercise and rIPC in cSVD patients. In contrast with our findings, previous studies reported an upregulation of Flt3L and the Flt3L-system after a single bout of whole-body exercise in healthy subjects and patients with multiple sclerosis (Bonsignore et al., 2002; Zaldivar et al., 2007; Deckx et al., 2015). These different outcomes may relate to the timing of the blood draws after exercise (immediate vs. 1 h post), study populations and type of exercise; whole-body cycling/running exercise vs. local handgrip exercise. Such differences in exercise volume and intensity, with whole-body exercise also likely activating multiple organ systems, may contribute to the conflicting observations.

We also found a decrease in FGF-21 following handgrip exercise and rIPC. In line with Flt3L, FGF-21 may play a role in CVD. For example, FGF-21 infusion resulted in a lack of protection against I/R injury was observed in obese, but not healthy, rodent hearts (Patel et al., 2014). Similarly, other studies found that FGF-21 signaling is affected during cardiovascular disease (Wu et al., 2020). This suggests an altered FGF-21 signaling during cardiovascular disease, which might also occur in sCVD patients. Studies examining the acute effects of exercise on FGF-21 have presented conflicting results, ranging from an increase of FGF-21 following whole-body exercise in healthy individuals (de Sousa et al., 2021; Domin et al., 2021; Jurimae et al., 2021), small FGF-21 changes in obese subjects and even lower FGF-21 in older men (Taniguchi et al., 2016; Khalafi et al., 2021). The local handgrip exercise bout performed in our study makes it difficult to compare our results with these previous studies that have typically adopted whole-body exercise. Taken together, our results suggest that changes in Flt3L and FGF-21 may play a role in the effects of exercise and rIPC, but follow-up measurements would be required to better understand the link between these proteins and protection against I/R-injury.

Despite the overlap in inflammatory proteins between handgrip exercise and rIPC, especially those with the strongest downregulation (i.e., Flt3L and FGF-21), we also found differences between interventions in five proteins (i.e., TRANCE, CCL19, CCL11, CXCL5, and CD6). This suggests that handgrip exercise has distinct effects on some proteins compared to rIPC. Although none of these differences remained significant after adjustment for multiple testing, these findings may suggest that the effects of handgrip exercise and rIPC, at least partly, work through distinct pathways.

*Limitations.* Our study also has some limitations. An obvious limitation is that we did not implement a control condition, which makes it difficult to directly relate the changes over time we found to either handgrip exercise or rIPC. The changes in inflammatory biomarkers may be attributed to the time of day, especially since inflammatory markers (e.g., FGF-21) fluctuate during the day (Bookout et al., 2013). However, no such evidence for a circadian rhythm of Flt3L has previously been reported. Future studies are warranted to explore whether handgrip exercise can also be translated to clinically meaningful effects in humans. Secondly, we cannot rule out that different exercise protocols could alter the magnitude of the effect compared to handgrip exercise. We adopted a handgrip exercise protocol, rather than whole-body exercise, because it allows for a more valid comparison with the local rIPC stimulus. Whole-body exercise has a wide variety of systemic effects (e.g., sympathetic nervous system, heart rate), which makes it difficult to compare exercise with rIPC. Moreover, handgrip exercise has potent clinical validity, since handgrip exercise will be easy to translate to clinical situation, including patient populations. A previous study showed that high-intensity cycling exercise protects against arterial dysfunction after prolonged ischemia, whereas moderate intensity exercise did not provide this same protective effect (Seeger et al., 2015). Similarly, the handgrip intervention performed in this study adopts an intermittent protocol of higher intensity exercise of the forearm muscles. One may question whether the magnitude of protection can be further enhanced by increasing the intensity and/or the volume of active muscle mass. In contrast, other studies showed that already moderate-intensity exercise can trigger preconditioning-related pathways (Thijssen et al., 2018). These studies suggest that presence of local (muscle) ischemia during exercise is not obligatory for the potential preconditioning effects. At least, our study is the first to demonstrate that even handgrip, activating a relatively small number of muscles, is sufficient to influence inflammatory protein expression that could potentially be signaling molecules associated with preconditioning.

In conclusion, single handgrip exercise performed by people with cSVD immediately attenuated plasma Flt3L and FGF-21, which seems comparable with the effects of rIPC. The reduction of Flt3L remains present across 4 days of repeated handgrip exercise. Flt3L and FGF-21 may both serve as biomarkers for these preconditioning interventions. Our data suggest that there is partial overlap in the inflammatory pathways activated by a single and repeated (4-day) exposure to local handgrip and upper arm ischemic preconditioning stimuli in patients with cSVD.

## Data Availability

The datasets presented in this study can be found in online repositories. The names of the repository/repositories and accession number(s) can be found in the article/Supplementary Material.
